# Highly Efficient Semi-Continuous Extraction and In-Line Purification of High β-O-4 Butanosolv Lignin

**DOI:** 10.3389/fchem.2021.655983

**Published:** 2021-05-10

**Authors:** Douwe Sjirk Zijlstra, Joren de Korte, Ernst P. C. de Vries, Lisanne Hameleers, Erwin Wilbers, Edita Jurak, Peter Joseph Deuss

**Affiliations:** ^1^ Department of Chemical Engineering (ENTEG), University of Groningen, Groningen, Netherlands; ^2^ Department of Bioproduct Engineering (ENTEG), University of Groningen, Groningen, Netherlands

**Keywords:** lignin, biomass, organosolv extraction, flow-through extraction, high β-O-4 content, carbohydrate removal

## Abstract

Innovative biomass fractionation is of major importance for economically competitive biorefineries. Lignin is currently severely underutilized due to the use of high severity fractionation methodologies that yield complex condensed lignin that limits high-value applicability. Mild lignin fractionation conditions can lead to lignin with a more regular C-O bonded structure that has increased potential for higher value applications. Nevertheless, such extraction methodologies typically suffer from inadequate lignin extraction efficiencies and yield. (Semi)-continuous flow extractions are a promising method to achieve improved extraction efficiency of such C-O linked lignin. Here we show that optimized organosolv extraction in a flow-through setup resulted in 93–96% delignification of 40 g walnut shells (40 wt% lignin content) by applying mild organosolv extraction conditions with a 2 g/min flowrate of a 9:1 n-butanol/water mixture with 0.18 M H_2_SO_4_ at 120°C in 2.5 h. 85 wt% of the lignin (corrected for alcohol incorporation, moisture content and carbohydrate impurities) was isolated as a powder with a high retention of the β-aryl ether (β-O-4) content of 63 linking motifs per 100 C9 units. Close examination of the isolated lignin showed that the main carbohydrate contamination in the recovered lignin was butyl-xyloside and other butoxylate carbohydrates. The work-up and purification procedure were investigated and improved by the implementation of a caustic soda treatment step and phase separation with a continuous integrated mixer/separator (CINC). This led to a combined 75 wt% yield of the lignin in 3 separate fractions with 3% carbohydrate impurities and a very high β-O-4 content of 67 linking motifs per 100 C9 units. Analysis of all the mass flows showed that 98% of the carbohydrate content was removed with the inline purification step, which is a significant improvement to the 88% carbohydrate removal for the traditional lignin precipitation work-up procedure. Overall we show a convenient method for inline extraction and purification to obtain high β-O-4 butanosolv lignin in excellent yields.

## Introduction

**FIGURE 1:**
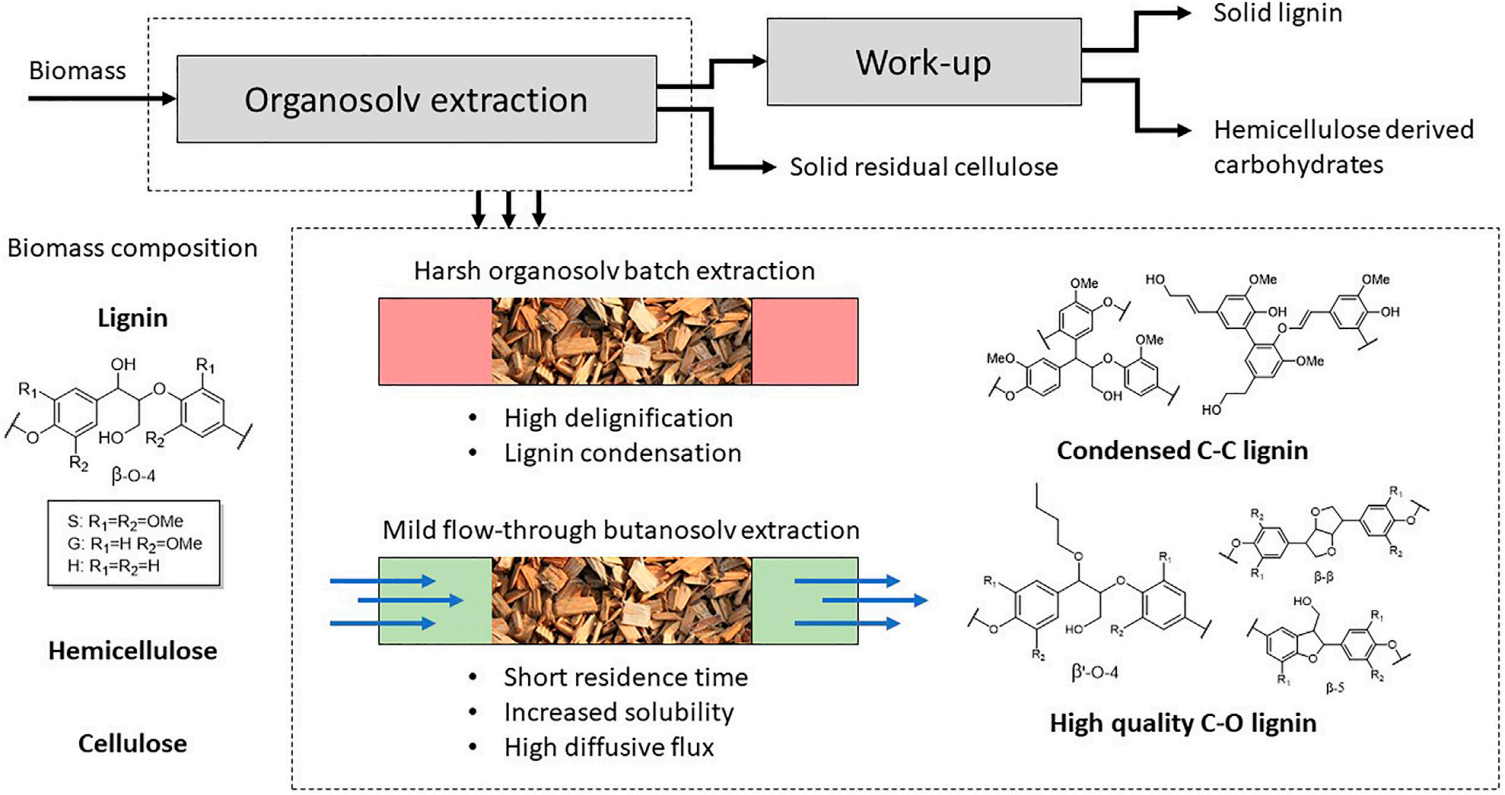
Schematic overview of mild organosolv fractionation of biomass and the influence of the extraction conditions.

In recent years there has been a focus on the production of chemicals from non-edible lignocellulosic biomass residues in order to replace current fossil feedstocks. These biorefineries rely on maximum value-extraction from all biomass components in order to be able to compete with existing industries (Ragauskas et al., 2006; Sheldon, 2014; Alonso et al., 2017). One of the currently under-utilized components is lignin which constitutes of around 15–40% of the dry mass of lignocellulosic biomass (Yoo et al., 2020). Lignin is an aromatic polymer consisting of substituted propyl phenol units of the types p-hydroxyphenyl (H), guaiacyl (G), and syringyl (S), which would make it a great replacement for aromatic chemicals (Figure 1) (Laurichesse and Avérous, 2014; Li et al., 2015). A complicating factor is the variety of linking motifs in lignin that are a result of the biosynthesis that includes a radical pathway (Ralph et al., 2019). Nevertheless, most aromatic units are linked via the β-O-4 motif (50–90%) shown in Figure 1. Due to its high abundance it is the prime focus of many novel catalytic conversion methodologies that either focus on the depolymerization of the single C-O bond or selective modification of the aliphatic -OH groups present in the β-O-4 motif to open new depolymerization pathways, introduce new functionality or enhance the solubility to enhance the processability of lignin (Sturgeon et al., 2014; Xu et al., 2014; Deuss et al., 2017; Sun et al., 2018; Wang and Wang, 2019) (Rahimi et al., 2013; Dabral et al., 2018; Magallanes et al., 2019) (Panovic et al., 2017, 2019b; Sameni et al., 2017; Zhang et al., 2019, 2020; De Santi et al., 2020; Zijlstra et al., 2020). However, the first step in lignocellulose processing is often the separation of the major components; lignin, cellulose and hemicellulose. Depending on the applied conditions the β-O-4 motif and the structure of lignin can be severely affected (Schutyser et al., 2018; Xu et al., 2020; Yoo et al., 2020).

Organosolv extraction is a methodology for the separation of the three lignocellulose components in relative pure fractions (Figure 1). In an ideal scenario all three components are effectively implemented into high-value applications. The C_5_ and C_6_ sugars can be used for fermentation or converted to any of the sugar derivable building block chemicals (1,3-diacids and FDCA) and lignin can be selectively depolymerized to aromatic building blocks (Isikgor and Becer, 2015). However, typically harsh extraction conditions are applied to obtain high yield of isolated lignin, which also lead to undesired condensation reactions and breakdown of the highly abundant β-aryl ether unit (Toledano et al., 2013; Huijgen et al., 2014; Wang et al., 2017; Hochegger et al., 2019; Hong et al., 2020; Liu et al., 2020; Meyer et al., 2020; Yang et al., 2020). This results in a highly complex new lignin structure with a higher C-C bonded structure compared to the C-O bonded native lignin. This is detrimental for the application into selective modification methodologies towards higher value application. Applying mild organosolv conditions yields lignin with a better retention of the C-O structure due to good retention of the β-aryl ether (Bauer et al., 2012; Zikeli et al., 2016; Lancefield et al., 2017; Zijlstra et al., 2020). This results in the isolation of a lignin which much closer resembles the structure of native lignin, but usually at the expense of a significant decrease in the lignin extraction efficiency.

Many mild organosolv extraction methodologies have been developed in the past years including the OrganoCat process (vom Stein et al., 2011; Weidener et al., 2018), stabilization strategies with aldehydes (Shuai et al., 2016; Chen et al., 2019) and the use of alcoholic solvents (Pan et al., 2007; Bauer et al., 2012; Huijgen et al., 2012; Wen et al., 2013; Zhang et al., 2016; Lancefield et al., 2017; Park et al., 2018; Tekin et al., 2018; Ramezani and Sain, 2019; Karlsson et al., 2020). The use of alcohol solvents under acidic extraction conditions results into incorporation of the alcohol into the β-aryl ether structure via α-alkoxylation by reaction with the benzylic cation, resulting in a new structure (denoted as β’-O-4, Figure 1) (Lancefield et al., 2017; Zijlstra et al., 2020). The incorporation of the alcohol into the lignin structure is beneficial for efficient extraction as it traps the reactive benzylic cation formed under acidic conditions reactions (Lundquist and Lundgren, 1972; Imai et al., 2011; Sturgeon et al., 2014; Deuss and Barta, 2016; Li et al., 2020). This prevents undesired condensation reactions and it allows the use of higher acid concentrations, which is beneficial for efficient extraction. Additionally, the incorporation of the alcohol into the lignin structure is highly beneficial for the extraction of in particular large lignin fragments (Zijlstra et al., 2020) as the Hildebrand solubility parameter of these lignin fragments is effectively decreased upon alkoxylation and thereby increases the solubility in organic solvents (Sameni et al., 2017). Especially for butanosolv extractions high lignin yields are reported (Lancefield et al., 2017; Panovic et al., 2019a; Zijlstra et al., 2020). This is likely a result of the high degree of alcohol incorporation when n-butanol is applied as extraction solvent, resulting in a modified lignin structure with enhanced solubility in organic solvents. The use of alcoholic solvents under acidic condition does not only modify the lignin structure, but also results in modification of the carbohydrates. The formation of alkoxy-xylosides has been extensively reported for sulfur dioxide-alcohol-water (SAW) fractionation (Iakovlev and Van Heiningen, 2012; Sharazi and Van Heiningen, 2017; Sharazi et al., 2017, 2018) and also for organosolv extractions (Lancefield et al., 2017).

The majority of the work on lignin extractions focuses on batch extractions, but in the past years there has been an increasing focus on (semi-)continuous extractions applying aqueous acidic and organosolv extraction conditions (Laskar et al., 2013; Zhang et al., 2015; Lohre et al., 2017; Løhre et al., 2017; Zhou et al., 2019a, 2019b; Tian et al., 2019; Wang et al., 2019; Majdar et al., 2020). Additionally, biomass fractionation has been reported for subcritical water flow through extractions (Pedras et al., 2017, 2020; Maciel-Silva et al., 2019). This methodology is particularly efficient for the recovery of carbohydrates, but also part of the lignin can be recovered with a high retention of the β-O-4 linking motif (Martínez-Abad et al., 2018). A reported extraction methodology with an initial subcritical water extraction with a sequential organosolv extraction showed that the bulk of lignin and carbohydrates can be removed effectively (Muniz Kubota et al., 2018). Flow extractions have some significant advantages over batch extractions that warrant this interest. The most significant advantage with regard to the extraction of lignin with a high structural quality is a lower residence time. Lignin suffers from degradation reactions at most extraction conditions following extraction due to a combination of the elevated temperature and acidic environment. This was nicely illustrated by following a 2 h methyl isobutyl ketone/ethanol/water extraction with sulfuric acid as catalyst at 140°C by sampling every 15 min. The number of phenolic hydroxyl groups doubled during the extraction and a corresponding decrease in aliphatic hydroxyl groups was observed, clearly showing the increased number of condensed structure over time (Tao et al., 2016). The application of flow setups that quickly remove the extracted lignin are therefore highly beneficial as this greatly reduces the time lignin is exposed to the harsh conditions extraction conditions, considerably decreasing the degree of condensation in the isolated lignin (Zijlstra et al., 2019a; Zhou et al., 2019b; Wang et al., 2019). Conversely, flow extractions can be applied at harsher conditions resulting in better extraction efficiency with a limited decrease in structural quality. With regard to efficient extraction flow setups have an additional advantage that fresh solvent is added continuously to the system. The diffusive flux remains therefore high throughout the entire extraction, whilst batch extractions suffer from decreasing diffusive flux as the lignin concentration increases over time. As the diffusion of dissolved lignin molecules is the rate-limiting step in the delignification process, this will enhance the extraction efficiency (Anderson et al., 2018; Kawamata et al., 2019). Furthermore continuous operation is highly desirable from an industrial point of view. The major disadvantage of flow setups is the high solvent consumption, which is even more profound for lignin extractions as these are typically not solvent efficient (Renders et al., 2019). High lignin extraction efficiencies are therefore required for flow setups to make them economically viable. The desired high extraction efficiency was reported for a 63/37 ethanol/water extraction at 175°C, yielding a 86% extraction efficiency for a 10 h flow extraction (Lohre et al., 2017). Recent work has shown the positive effect of the short residence in a flow set-up on the structural quality of the isolated lignin. For both aqueous formic acid extractions at 130°C (Zhou et al., 2019b; Tian et al., 2019) and p-toluenesulfonic acid at 70–98°C (Wang et al., 2019) retention of over 30 β-O-4 linking motifs was reported, whilst comparable batch extractions showed a significant decrease to <10 β-O-4 linking motifs.

Organosolv extractions can also applied in combination with reductive fractionation, which depolymerizes lignin into alkyl-phenolics in high yield (Van den Bosch et al., 2015; Huang et al., 2017; Liao et al., 2020). There is an incentive to move towards flow-through reactors for reductive fractionation (Anderson et al., 2017, 2018; Kumaniaev et al., 2017), which compared to batch offers greater flexibility as the process conditions can be changed over the course of the reaction effectively (physically) separating the extraction/(solvolytic) depolymerization step from the (hydrogenolysis) depolymerization/stabilization step (Renders et al., 2019). This increased focus on flow-through reductive fractionation highlights the importance of an effective flow extraction that yields high purity lignin with high retention of the β-O-4 content to allow for efficient depolymerization in the subsequent step.

Previous work in our laboratory with a flow-through setup focused primarily on ethanosolv extractions at 120°C (Zijlstra et al., 2019a). This study showed a clear increase in both quality (high retention of β-O-4 content, 62 β-O-4 linking motifs), and quantity (improved yield, 57% extraction efficiency) compared to traditional ethanosolv batch extractions at similar conditions. This was attributed to a lower residence time of the extracted lignin at reactive conditions and the continuous introduction of fresh solvent, resulting in a higher diffusive flux in the flow-through setup. Surprisingly the flow-through setup did not necessarily lead to a higher efficient solvent usage compared to batch extractions performed at identical conditions. The solvent efficiency was typically in the range of 20 mg lignin extracted per gram of solvent used, which was similar for batch extractions. Work with different alcohols showed that propanosolv and in particular butanosolv might provide further improvements, but appropriate comparison was somewhat limited by low solubility of water in butanol. Additionally, 2D HSQC NMR analysis of butanosolv showed the presence of contaminants which were absent in ethanosolv lignin. Based on these results, we aimed to further investigate the potential of butanosolv extraction in a flow-through setup the development of a convenient (in-line) work-up procedure, with the overall goal to develop a procedure to obtain butanosolv lignin in high yield with a high quality (high β-O-4 content) and purity (limited carbohydrate impurities).

## Experimental

### Materials

1-butanol (99% purity) and acetone-d_6_ (99.5 atom% D) were purchased from Acros organics. Silicon dioxide (fused granular, 99.9% purity, mesh 4–20), sodium sulfate (99.0% purity), sodium hydroxide (≥97.0% purity), deuterated chloroform (99.8 atom% D), chromium acetylacetonate (97% purity), 2-chloro-4,4,5,5-tetramethyl-1,3,2-dioxaphospholane (95% purity), and deuterium oxide (99.9% atom% D) were purchased from Merck. Sulfuric acid (95–97% purity) and tetrahydrofuran (stabilized with BHT) were purchased from Boom B.V. Acetone (99.5% purity) and toluene were purchased from Macron Fine Chemicals. Endo-hydroxy-5-norbornene-2,3-dicarboximide was purchased from Fisher scientific. Walnut powder was bought from Brambleberry (Bellingham, WA, United States). Biomass preparation was performed via an earlier published procedure (Zijlstra et al., 2019b), which includes ball-milling in order to obtain small particles and the removal of extractives from the biomass by a 2 h toluene extraction at reflux conditions in a roundbottom flask. The extraction liquor was removed by filtration and the extractive-free lignocellulosic feedstocks were dried in an oven in vacuo for 16 h at 70°C.

### Experimental Procedure

**Butanosolv extraction:** A flow-through reactor of 100 mL (Supplementary Figure S3) was loaded with biomass (typically 40.0 g walnut powder, moisture content 5 wt%, composition Supplementary Tables S1–S3). Previous ethanosolv extractions did not show a difference in extraction efficiency for the different particle size fractions. The granules, biomass, and glass filters were separated with quartz wool. A 9:1 volumetric solvent mixture of 1-butanol/water with either a 0.1 M or 0.18 M H_2_SO_4_ concentration was pumped through the reactor with a speed of 2 g/min (retention time ∼30 min) for a total of 2.5 h. The flow speed was manually adjusted and determined by measuring the weight of the outflow stream. The setup is positioned so that the solvent enters the reactor from the bottom and leaves the reactor at the top. The reactor is filled with the extraction liquid (with acid) and the pressure was increased to 6 bar to ensure that the solvents remain in the liquid phase. When the desired pressure was obtained (with a constant outgoing flow), the system was heated to 120°C. The start of the experiment (*t* = 0) was set as the moment the temperature reached 100°C, as previous work has shown that the first relevant extractables exit the reactor from that point onwards. The extraction liquor was evaporated in vacuo by rotary evaporation. The obtained solid was redissolved in a minimal amount of acetone and 2 mL water and precipitated in a 500 mL dilute aqueous solution of sodium sulphate (400 mL water + 100 mL saturated aqueous sodium sulphate solution). Sodium sulphate was essential as it enhances flocculation of the product. Absence of sodium sulphate results in a sticky black product, which can not be effectively recovered by filtration and requires redissolving and a second precipitation step in order to obtain a recoverable product with lower yield. Additionally, full removal of the n-butanol is required during the concentration step, as the presence of n-butanol results in the formation of an emulsion and results in inefficient precipitation. The product was obtained by filtration, washed with an additional 50 mL of water and dried under an air-flow. The precipitated and air-dried lignin was used for the determination of the extraction efficiency and the structural quality.

**Biphasic purification:** A continuous integrated mixer/separator (CINC) was applied for the mixing and separation of the different phases (Supplementary Figure S1). The two inlet streams are combined in the mixed part at the bottom of the reaction chamber (6000 rpm). Travelling up the centrifugal cone, the heavy phase gets propelled towards the outer edge. Due to the design of the CINC the heavy phase is discharged from the outlet at the top and the lighter fraction is obtained from the outlet below it. One of the inlet streams is the untreated extraction liquor and can be connected directly with the flow-through reactor but also with a flask with collected extraction liquor. The other inlet stream is used to add either caustic soda (pH 13) or a saturated aqueous sodium sulphate solution with a similar flow to the incoming extraction liquor. The obtained butanol phase (light phase) and the residual mixture in the CINC are concentrated in vacuo and the obtained product is analysed both prior to and after the precipitation step as described in the traditional workup procedure. When caustic soda was used, the water phase was collected and sulfuric acid was reintroduced, resulting in the precipitation of lignin. A schematic representation is given in Supplementary Figure S4 All precipitated lignin is collected by filtration and air-dried under an air-flow.

**Carbohydrate impurity quantification:** The percentage of carbohydrate impurities present in the isolated lignin samples was determined on the intensity of the butyl-xyloside signals at (4.20,104.2) and (4.68,99.7) as these signals correspond to the two anomers present and these signals are in a distinct region in the 2D HSQC NMR spectra, with no overlap with lignin peaks. The sum of these two signals is used as the intensity of a single proton value of the xylose present in the sample. The other signals in that region are assumed to be corresponding to other anomeric carbohydrate moieties. The single proton value for lignin is based on the sum of the signal intensities of the three aromatic groups divided by the number of aromatic protons of each aromatic structure. The molecular weight of butyl-xyloside is 206 g/mol and the average molecular weight of a single monolignol unit is in the proximity of 250 g/mol, with the precise value depending on both the S/G/H ratio and the degree of alcohol incorporation. The calculation of the exact value has been performed according to literature (Zijlstra et al., 2020). With these values the amount of Carbohydrate impurities can be calculated by the following formula:

$$\begin{array}{l} \%\mathrm{carbohydrate impurities}\\ =\frac{\mathrm{carbohydrate fraction}*\mathrm{Mw carbohydrate}}{\mathrm{carbohydrate fraction}*\mathrm{Mw carbohydrate}+\mathrm{Lignin fraction}*\mathrm{Mw Lignin}}\;\times \mathrm{100\%} \end{array}$$

## Results and Discussion

### Butanosolv Extraction Optimization

This work focused on the optimization of our previously developed flow-through organosolv extraction set-up and conditions (Zijlstra et al., 2019a). This previous study showed that a 4:1 alcohol/water ratio was optimal for the extraction of ethanosolv walnut shell lignin under acidic conditions (pH ≈ 1) which allows for improved lignin and hemicellulose solubility. For both ethanol and n-propanol good lignin extraction efficiencies (52 and 57%) and structural quality (62 and 66 β-O-4 linking motifs per 100 C9 units) were achieved in 5 h extractions with a 1 g/min flowrate on 20 g walnut shells at 120°C. However, as the use of a 4:1 butanol/water ratio leads to phase separation, n-butanol could not be tested under the same conditions. The addition of 1,4-dioxane (80:15:5 n-butanol/H_2_O/1,4-dioxane) led to a single phase and the extraction with this solvent mixture resulted in a high extraction efficiency of 74%. However, the structural quality decreased significantly to 54 β-O-4 linking motifs per 100 C9 units (accompanied with the corresponding condensation of aromatic units) which was mainly caused by the lignin isolated past 2 h extraction time. Control experiments with ethanol and n-propanol in the presence of 1,4-dioxane showed a clear negative influence on the structural quality (an average decrease of 10 β-O-4 linking motifs) of the isolated lignin by the 1,4-dioxane addition. For this reason, we aimed to further investigate the butanosolv lignin extraction without 1,4-dioxane addition.

Water and n-butanol form a single phase at a water content of 16% or lower. As previous work showed that decreasing the water ratio as low as 5% had a negative influence on ethanosolv extractions (Zijlstra et al., 2020), a 9:1 n-butanol/H_2_O v/v ratio was chosen as a starting point for this work. Additionally, the acidity was decreased by lowering the sulfuric acid concentration from 0.18 to 0.10 M and the flow rate was doubled to a 2 g/min flow rate (and extraction time halved) to reduce the residence time, limiting the engagement of the extracted lignin in secondary reaction (Lancefield et al., 2017). Both of these changes were introduced as an effort to further reduce the degree of condensation (associated with the loss of β-O-4 content) in the obtained product.

**FIGURE 2:**
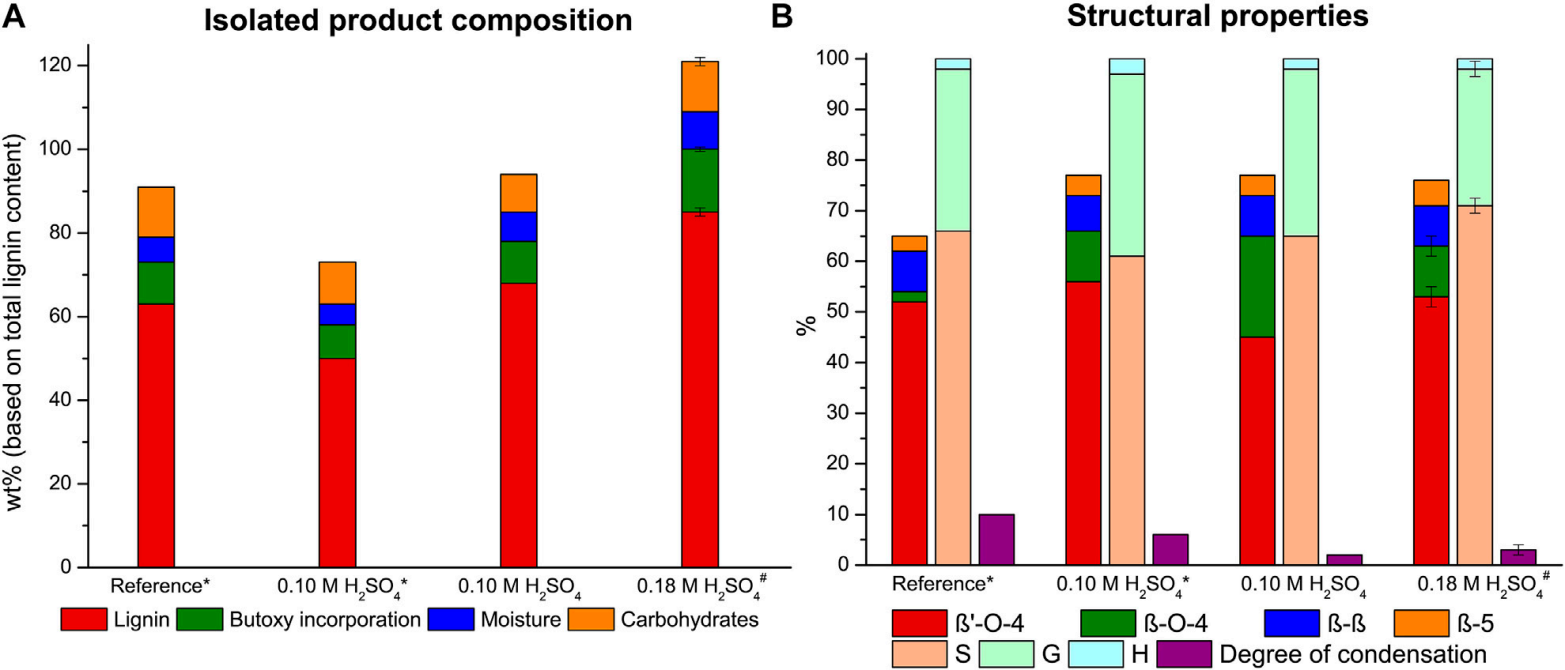
Lignin extraction efficiency with product composition with each component expressed as a percentage of the total lignin content **(A)** and the linkage content (per 100 C9 units), S/G/H ratio and the degree of condensation (based on condensed S-units) **(B)** of the isolated butanosolv lignin for the reference extraction (80:15:5 nBuOH/H_2_O/1,4-dioxane, 0.18 M H_2_SO_4_) and the optimization study with a 9:1 nBuOH/H_2_O ratio. * half reactor loading # average of two runs.

We had previously noted the importance of concise analysis of the lignin for accurate yield determination. In our previous work we already corrected lignin recovery masses for alcohol incorporation (up to 20% added weight to lignin for full n-butanol incorporation). However, we noticed that it is important to consider moisture content (up to ∼10 wt%, determined with a PCE-MA 110 moisture meter (method in SI)) and in particular for butanosolv extractions the carbohydrate impurities should be considered as these also have a significant contribution to the mass of the isolated product (up to ∼12 wt% see later detailed discussion)*.* Therefore, all the reported values for lignin yields throughout this work are corrected for these three values to allow for a proper discussion in terms of the fate of the lignin. This correction was also applied to our previously reported 80:15:5 n-butanol/H_2_O/1,4-dioxane extraction efficiency value (reference extraction, Figure 2), which was corrected to a 63% extraction efficiency (from 74%) due to the significant amount of carbohydrate impurities and this value will be now referred to as the lignin recovery (Figure 2A).

The extraction at the aforementioned adjusted conditions was performed with 20 g walnut powder and resulted in a reduced lignin recovery of 50%. Nevertheless, the β-O-4 content of the isolated lignin was very high with 66 β-O-4 linking motifs per 100 C9 units (Figure 2B). The structural quality of this butanosolv lignin is in correspondence with the highest reported values for the β-O-4 content of lignins extracted from walnut shells (Lancefield et al., 2017; Zijlstra et al., 2020) and in correspondence with the β-O-4 content of milled wood lignin and enzymatic hydrolysis lignin obtained from the same biomass source (Supplementary Table S2). Additionally, a small decrease in both the degree of condensation (from 10 to 6%) and degree of alcohol incorporation (from 96 to 85%) were observed compared to the reference conditions. These were attributed to a combined effect of both a shorter retention time and the decreased acid concentration.

Our previous work on semi-continuous ethanosolv extractions showed that higher reactor loading was beneficial for the extraction efficiency (Zijlstra et al., 2019a). Indeed, performing the extraction with 40 g walnut powder (maximum reactor capacity) showed a lignin recovery increase to 67%, whilst maintaining the same structural quality of 65 β-O-4 linking motifs. The increase in extraction efficiency is attributed to the increased (double) contact time between the solvent and the feedstock. This is due to the use of a tubular reactor with a small diameter in the extraction setup. With an increased reactor loading the biomass level in the reactor increases, resulting in an increased contact time (Supplementary Figure S2). This increase in extraction efficiency with longer contact time strongly implies mass transfer limitations for the lignin extraction at the applied extraction conditions. With a full reactor loading the product loss during work-up is relatively smaller compared to small scale extractions. Quite notably less condensation product (2%) was observed and the degree of alcohol incorporation showed a small decrease to 69% for full reactor loading. We think that due to the lower solvent to biomass ratio less benzylic cations are formed by the acid relative to the total number of lignin β-O-4 linking motifs leading both to a decreased amount of alcohol incorporation and degradation reactions (Lundquist and Lundgren, 1972; Imai et al., 2011; Sturgeon et al., 2014; Deuss and Barta, 2016). Additionally, with a full reactor loading, part of the lignin is extracted closer to the reactor outlet when the biomass bed is higher. This results in a shorter average exposure of the extracted lignin to the extraction conditions and therefore less time for secondary reactions like condensation reactions, as was also observed by zeolite induced β-O-4 cleavage (Kramarenko et al., 2021).

The extraction efficiency could be further improved by increasing the acid concentration to 0.18 M sulfuric acid, which resulted in a very high lignin recovery of 85% (average of 2 runs, 86 and 84% lignin recovery respectively) accompanied by a small decrease in β-O-4 content to 63 linking motifs per 100 C9 units. This optimized extraction condition was also applied for the in-line purification experiments *vide infra* and yielded an average lignin recovery of 74% (average of 4 runs) of high-purity butanosolv lignin, showing a constant reproducibility of the high extraction efficiency and structural quality. Despite the increase in acidity, the degree of condensation showed only a minor increase to 3%. Analysis of the residual biomass of the two highly efficient butanosolv extractions showed a residual lignin content of 4 and 7% of the original lignin content respectively, showing that almost full delignification can be achieved with these relatively mild extraction conditions. The good lignin recovery at these mild conditions are attributed to the improved solubility of butoxylated lignin due to the *a*-alkoxylation in an acidic environment that we previously showed occurs already on the non-solubilized lignin (Zijlstra et al., 2020). The benefit of this improved solubility is combined with a low residence time of the extracted lignin in this flow-through setup minimizing the potential of secondary reactions. One of the typical downsides of flow extraction is the high solvent consumption relative to the amount of product extracted. With the optimized conditions a solvent efficiency of 50 mg lignin per gram of extraction solvent was achieved. A wide range of lignin extraction procedures yielding lignin with at least a moderate retention of the β-O-4 linking motif were analyzed, showing a solvent efficiency in the range of 9–47 mg per Gram of solvent (Supplementary Table S4). (Bauer et al., 2012; Wildschut et al., 2013; Shuai et al., 2016; Lancefield et al., 2017; Smit and Huijgen, 2017; Wang et al., 2017; Ouyang et al., 2018; Sharazi et al., 2018; Weidener et al., 2018; Dong et al., 2019). This showed that the flow-through methodology was very efficient in terms of solvent efficiency, especially considering the very high structural quality of the isolated lignin. Even though it is quite hard to properly compare extractions with different feedstocks, this clearly illustrates the excellent potential for flow-through organosolv extraction with butanol/water as extraction mixture.

**FIGURE 3:**
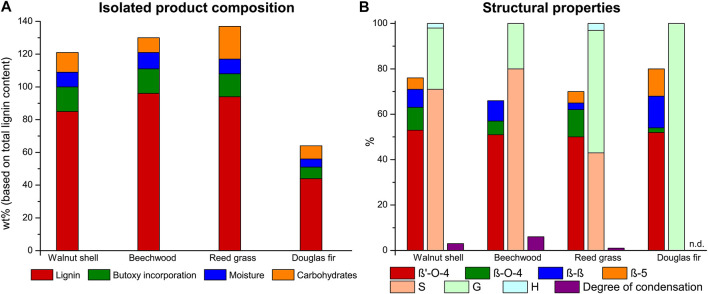
Lignin extraction efficiency with product composition with each component expressed as a percentage of the total lignin content **(A)** and the linkage content (per 100 C9 units), S/G/H ratio and the degree of condensation (based on condensed S-units) of the isolated butanosolv lignin **(B)** obtained with the optimized extraction conditions (9:1 n-butanol/water, 0.18 M H_2_SO_4_) on different feedstocks.

Applying the optimized extraction conditions to beechwood and reed grass (structural composition given in Supplementary Table S1) showed good to excellent lignin recovery (96 and 94%, Figure 3A) in combination with a high retention of the β-O-4 linking motifs (57 and 62 β-O-4 linking motifs, respectively, Figure 3B). This shows that this butanosolv extraction methodology can readily be extended beyond walnut shells to other lignocellulosic feedstocks as was previously shown in batch (Balogh et al., 1992; Teramura et al., 2016; Lancefield et al., 2017; Panovic et al., 2019a; Liu et al., 2019; Foltanyi et al., 2020). Nevertheless, softwoods proved more recalcitrant with 44% lignin recovery for douglas fir (54 β-O-4 linking motifs). This is similar to a reduction of yield for butanosolv extraction going from beech and walnut to douglas fir in batch (Lancefield et al., 2017). Furthermore, extraction of pine wood resulted in the isolation of a product with significant yield but upon analysis showing few lignin motifs revealing something that was more in line with a suberinic type of material (Supplementary Figure S12 (Karnaouri et al., 2016)). Extraction conditions likely need to be further tuned for these specific feedstock types. Analysis of the residual material showed that this organosolv procedure is not only efficient for delignification, but also efficiently extracts the majority of the hemicellulose (Supplementary Table S1). Carbohydrates that make up the branches (galactose and arabinose) are fully removed at the applied extraction conditions, as is the bulk of the main chain (xylose and mannose).

### Lignin Recovery/Purification

**FIGURE 4:**
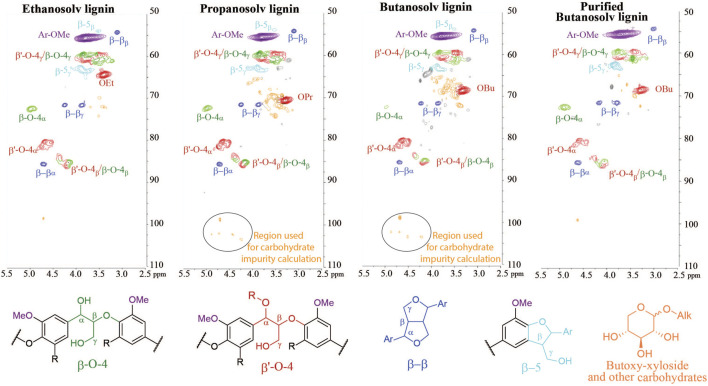
2D HSQC NMR spectra {[(2.5–5.5)(50–110)] ppm linkage region} of ethanosolv, propanosolv, and butanosolv lignin obtained by the traditional work-up method and purified butanosolv lignin isolated with a caustic soda treatment, showing a significant reduction in the carbohydrate content.

Lignin is typically recovered as a solid from an organic extraction phase by water precipitation. In the precipitation step, the concentrated extraction liquor is added to an excess of water leading to a precipitate that can be filtered off. Butanosolv lignin is notoriously more difficult to precipitate than ethanosolv and propanosolv lignin and the addition of sodium sulfate to the water is absolutely necessary to enhance flocculation of butanosolv lignin (Supplementary Figure S6 clearly shows the influence of sodium sulfate). Furthermore, n-butanol has to be completely removed during the concentration step and the concentrated butanosolv extraction liquor can only be added very slowly to a vigorously stirred aqueous solution, otherwise a sticky solid material is obtained. Even with this improved work-up, close analysis of the 2D HSQC NMR spectra of the isolated butanosolv lignin samples showed signals in the region [(3.0–4.0)(60–80)] ppm and a few distinct signals in the region [(4.0–5.0)(90–110)] ppm which could not be attributed to lignin linking motifs. Such signals are typically absent for ethanosolv lignin and present to a much smaller extent for propanosolv lignin (Figure 4). Similar impurities could be observed in other reported work on butanosolv extractions (Lancefield et al., 2017; Panovic et al., 2019a).

Lancefield and co-workers reported the formation of butyl-xyloside and other butoxylated carbohydrates at the given extraction conditions, which were identified by ^13^C-NMR analysis of the water layer obtained during the precipitation step (Lancefield et al., 2017). Butyl-xyloside has two distinct peaks at (δ 4.20, δ 104.2) and (δ 4.68, δ 99.7) in 2D HSQC NMR that correspond to the two anomeric protons and an overlay with the butanosolv lignin impurities showed that the majority of the impurities likely stem from alkoxylated carbohydrates (SI). These seem to be inadequately removed by the typical lignin precipitation step, which is likely due to their improved organic solvent solubility of alkyl-xyloside going from ethoxy to propoxy to butoxy. Indeed butyl-xyloside does not adequately dissolve in water leading to a suspension (Supplementary Figure S7). This confirms that butoxy-modified carbohydrates has indeed mediocre solubility in water and therefore partially precipitate together with the lignin.

To quantify the impurities in our isolated lignins, we decided to use the 2D HSQC NMR signals in the anomeric region (circled in Figure 4, formula shown in methods). Applying this formula on multiple butanosolv lignins showed the amount of carbohydrate impurities is consistently around 10–12 wt%, which is considerably higher than the values for ethanosolv lignin (up to max 2 wt%), and propanosolv lignin (around 4 wt%). It should be noted that butanosolv extractions are widely considered as the most efficient method for alcoholic solvents for lignin extraction solely based on the recovered mass (often not even corrected for the butoxy incorporation and the moisture content as we do here). As this result shows, the use of this recovered mass can lead to an overestimation of the isolation efficiency. More importantly, the presence of carbohydrate impurities in the isolated lignin could have a detrimental effect on the applicability of butanosolv lignin. Nevertheless, even with appropriate corrections, butanol still outperforms other alcohols for extraction at mild conditions and thus we were interested in a more appropriate work-up procedure to obtain butanosolv lignin free of carbohydrate impurities.

As the n-butanol/water ratio applied for the lignin extractions is close to the maximum solubility of water in butanol, purification by means of phase separation can be achieved with the addition of only small amounts of water. The direct addition of an aqueous saturated sodium sulfate mixture in a 1:1 ratio to the extraction liquor (without concentration) resulted in a clear phase separation between the water layer and the organic layer, although it took a considerable time before full separation was achieved. Subsequent work-up of the organic phase resulted in a lignin precipitation step that was noticeable easier with instant precipitation of the lignin. However, the 2D HSQC NMR analysis of the isolated lignin did not show an improvement in the purity (still about 12% carbohydrate impurities), indicating that the modified carbohydrates are not removed by this additional work-up procedure.

A different purification methodology on an isolated butanosolv lignin (63 β-O-4 linking motifs of which 80% correspond to the alcohol incorporated β’-O-4 linking motif) with 8% carbohydrate impurities was performed next. The butanosolv lignin was washed with caustic soda (pH 13) and stirred for 24 h at 50°C. Although part of the lignin becomes water soluble under basic conditions due to its high phenolic content, this lignin can be reprecipitated again by acidifying the mixture to neutral pH. Analysis of the recovered lignin showed the absence of carbohydrate impurities and additionally no decrease in structural quality and only a small decrease in alcohol incorporation (62 β-O-4 linking motifs of which 75% corresponds to the β’-O-4 linking motif). It was envisioned that under basic conditions partial de-alkoxylation of the carbohydrates would enhance the solubility of carbohydrates in water. Caustic soda treatment of butyl-xyloside indeed showed that the water phase contained an almost 1:1 ratio of xylose and butyl-xyloside, showing that part of the butyl-xyloside is reverted to xylose under these conditions and this explains the enhanced carbohydrate removal. Additionally, partial de-alkoxylation of the lignin could reduce the interaction with butoxy-carbohydrates and in combination with swelling of lignin by the alkali treatment this could enhance the release of trapped carbohydrates.

^31^P-NMR analysis of the butanosolv lignin could further confirm the removal of carbohydrates. With this method the individual aromatic -OH and aliphatic -OH content can be determined following derivatization with a phosphorus reagent (Constant et al., 2016). Upon caustic soda treatment of impure butanosolv lignin the ratio [aromatic -OH:aliphatic -OH] changed from 1:3.45 to 1:3.09 (Supplementary Table S5). A small degree of de-alkoxylation of butanosolv lignin was observed which is expected to lead to an actual increased aliphatic -OH content relative to the aromatic -OH content in a pure lignin sample. So, the observed decrease in the aliphatic -OH content fits well with the 8% carbohydrate removal as observed by 2D HSQC NMR spectroscopy. These results also highlight the large effect that carbohydrate impurities can have on the lignin -OH content determination by this methodology using ^31^P-NMR analysis.

These initial experiments showed that carbohydrates can be effectively removed using a caustic soda treatment. This procedure involves an extra operation with additional work-up steps that results in additional product loss and is too laborious and time consuming to perform in batch operation. Therefore, keeping in mind large scale lignin extraction, we were interested in investigating an integrated in-line caustic soda work-up for butanosolv lignin purification.

### In-Line Lignin Purification Using a Continuous Integrated Mixer/Separator (CINC)

**FIGURE 5:**
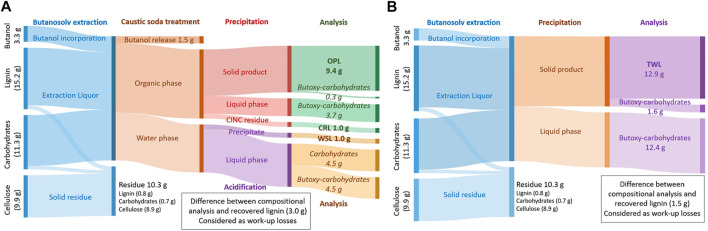
Sankey-diagrams of the lignin and polysaccharide fractions of a flow-through butanosolv lignin extraction of walnut shells with a combined in-line caustic soda treatment in a CINC device and subsequent precipitation step **(A)** and the traditional work-up procedure consisting of a single precipitation step **(B)**. Values between brackets are determined by compositional determination of the starting material and residue. OPL = Organic-phase lignin, CRL = CINC residual lignin, WSL = Water-soluble lignin, and TWL = Traditional work-up lignin.

To achieve a potential continuous extraction and work-up process, a continuous integrated mixer/separator (CINC, Supplementary Figure S1 (Schuur et al., 2012; Seyfang et al., 2019; Manavalan et al., 2020)) was implemented which was directly connected to the flow-through setup. Such a device can enhance extraction efficiency by enhanced mass-transfer due to the intensive mixing forces between the static housing. Furthermore, the rotating rotor allows for good phase-separation by the use of centrifugal forces where the heavy (water) phase is propelled towards the outer edge. In addition to the solvent flowing out of the extraction setup (from a 40 g biomass extraction) a second contact solvent is added to the CINC via a second inlet (pH 13 caustic soda). Both streams were added in a 1:1 ratio, each with a flowrate of 2 g/min and co-current mixing was applied at a 6000 rpm rotation speed. The overall lignin and carbohydrate mass streams (corrected for solvent incorporation and moisture content) from this extraction is shown in Figure 5A and for convenience compared to the mass flows of a traditional direct precipitation workup (Figure 5B).

**FIGURE 6:**
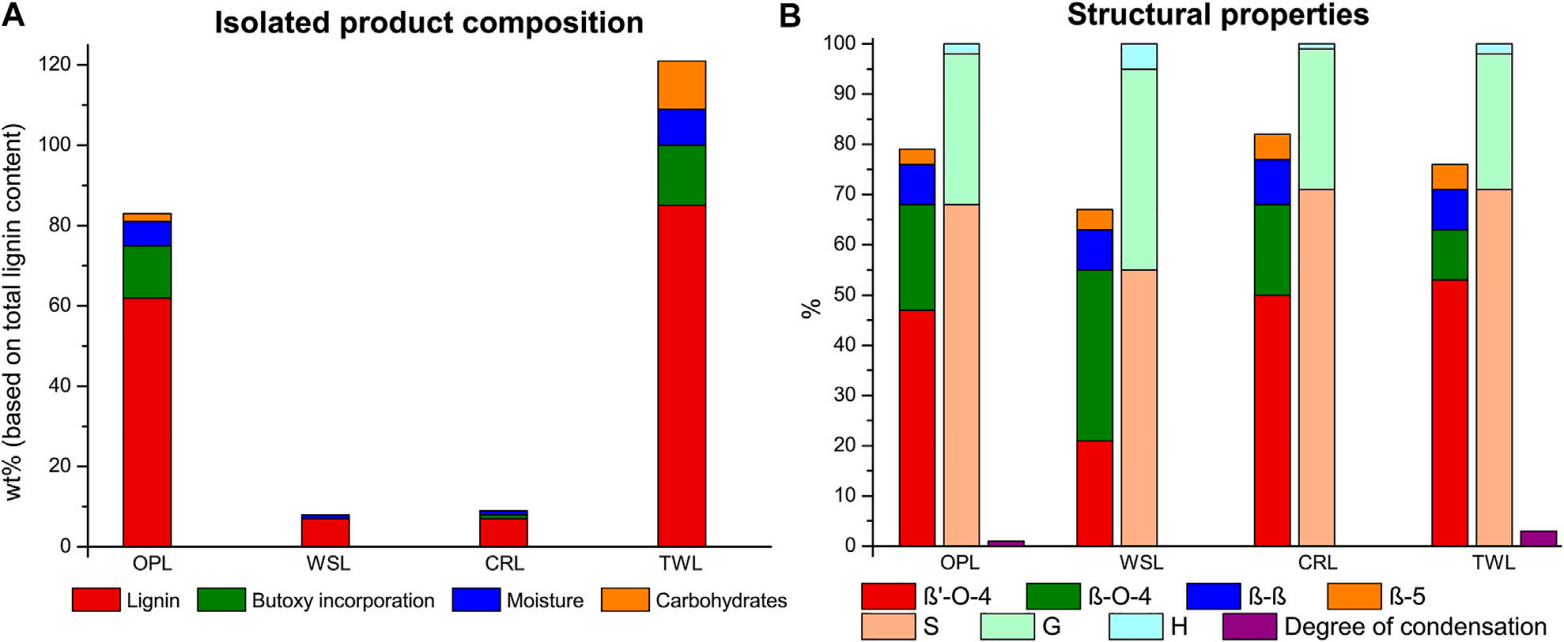
Lignin extraction efficiency with product composition with each component expressed as a percentage of the total lignin content **(A)** and the linkage content (per 100 C9 units), S/G/H ratio and the degree of condensation (based on condensed S-units) of the isolated lignin **(B)** for the organic phase lignin (OPL), water-soluble lignin (WSL), and CINC residual lignin (CRL) fractions obtained after caustic soda treatment and lignin obtained via the traditional work-up (TWL).

This in-line purification step with caustic soda on the extraction liquor resulted in the recovery of the major part of the lignin, as an average lignin recovery of 74% (4 runs) of the total lignin content of the biomass was achieved. Full analysis of all the streams showed that the majority of the lignin was recovered from the organic phase (OPL, 9.4 g, 62% of the total lignin content) and a small fraction of lignin could be recovered from the water phase (WSL, 1.0 g as a dark brown solid, 7% after acidification, Figure 6A). Additionally, not all lignin is recovered during the in-line purification step and some was found back after washing the CINC device (CRL, 1 g as a gray solid, 7%) for a combined total of 75% lignin recovery based on the lignin content of the biomass. The amount of carbohydrate impurities in OPL was 3% with a caustic soda washing step and subsequent precipitation, while the WSL and CRL were practically free of carbohydrate impurities. The impurities in OPL are significantly lower compared to the 12% carbohydrates impurities in TWL.

Further analysis of all the streams showed that the majority (69%) of the carbohydrates are effectively removed during the caustic soda treatment in the CINC (Figure 5). The carbohydrates are recovered as a mixture of carbohydrates and butoxy-carbohydrates. In the subsequent precipitation step almost all of the remaining carbohydrates (29%) are removed as only 0.3 g of carbohydrate impurities are present in the isolated lignin fraction, which corresponds to a combined carbohydrate removal of 98%. This is a significant improvement compared to the direct precipitation work-up of the extraction liquor in which 1.6 g of butoxy-carbohydrate impurities are present in the isolated lignin, which corresponds to a 88% carbohydrate removal. Overall, the lignin recovery from this in-line caustic soda was somewhat lower, but the fractions are significantly lower in carbohydrate impurities and precipitation was significantly easier.

Looking at the structural characteristics it can be seen that the caustic soda did lead to significant reduction of the butoxy modified β-O-4 linking motifs in all isolated fractions (Figure 6B). In-line with the results discussed in the previous section the caustic soda treatment seems to revert some β’-O-4 to β-O-4 units. Interestingly, the main OPL fraction actually had higher total β-O-4 content (β’-O-4 + β-O-4) compared to TWL. This can be because in the traditional work-up the extraction liquor is not neutralized during the concentration step, potentially causing some degradation of the β-O-4-motif. A caustic soda treatment can prevent any acid-catalyzed β-O-4 cleavage post-extraction during concentration at elevated temperature (typically 40–50°C under reduced pressure). Another contributing factor seems to be that smaller fragments (with inherently lower β-O-4 content and higher phenolic content) are more readily dissolved in water and thus end up in the WSL fraction. This was confirmed by molecular weight analysis which showed that WSL is significantly lower in MW compared to OPL (1900 and 3900 Da, Supplementary Figure S1 and Supplementary Table S6). Additionally, WSL contained a high H and G unit content, which are associated with end groups and thus with smaller fragments released from the larger native lignin polymer. Our previous work showed that a high degree of alcohol incorporation is required to obtain adequate solubility of large lignin fragments (Zijlstra et al., 2020). The decrease in alcohol incorporation due to the caustic soda treatment could result in lower solubility in the organic phase. This phenomena was further emphasized by looking at the CINC residual lignin (CRL) which consists of high molecular weight fragments (6900 Da, Supplementary Figure S11 and Supplementary Table S6). The high β-O-4 content combined with a reduction in butoxy incorporation could lead to large lignin fragments with lower solubility in both water and butanol which subsequently partly accumulate in the CINC. The color of the isolated lignin (Supplementary Figure S5) and the molecular weight are directly related to each other, as the color is significantly darker for low molecular weight lignin (WSL) compared to high molecular weight lignin (CRL). This attributed to the relatively high number of phenolics in short chain lignin fragments. These results show that, apart from efficient lignin purification, also some lignin fractionation occurs during the in-line caustic soda treatment mainly based on lignin molecular weight. It should be noted that fully continuous operation by for example coupling two parallel flow through extraction columns (e.g., used in work on reductive fractions (Anderson et al., 2017)), would likely effectively remove the CINC residual lignin stream, though an incidental washing step of the CINC (e.g., with THF in which these fragments are readily soluble) might be required to prevent the accumulation of large lignin fragments.

## Conclusion

High lignin extraction efficiency and the recovery of lignin with high linkage retention were achieved by flow-through butanosolv extractions of walnut shells (95% delignification and 63 β-O-4 linking motifs per 100 C9 units). This was achieved using an acidic 9:1 vol% n-butanol/water mixture (0.18 M sulfuric acid) and a 2.5 h extraction time with a 2 g/min flowrate on 40 g of walnut shells at 120°C. 85% of the lignin in the original biomass (corrected for alcohol incorporation, moisture content and carbohydrate impurities) was recovered as a precipitated solid. The general applicability of the extraction method was demonstrated on beech and reed giving even higher recovery of lignin with high structural quality. High quality lignin was also successfully extracted from douglas fir albeit at lower delignification, while pine showed poor results. Furthermore, the solvent efficiency proved to be excellent compared to batch extractions. Overall, this highly effective extraction method at mild conditions yielding high purity lignin shows that severe extraction conditions are not the only option if high delignification is required.

Full analysis of the isolated butanosolv lignins showed that, compared to other alcoholic solvent extractions, a significant amount of carbohydrate impurities (approximately 12%) was present, due to altered aqueous solubility of these carbohydrates by n-butanol incorporation into the chemical structure. An alternative work-up methodology was successfully introduced to reduce the carbohydrate contamination by adding caustic soda (pH 13) in a 1:1 weight ratio with the extraction liquor in a CINC, which was used for efficient mixing and phase separation of the water and organic layer. With this methodology a 75% recovery of high purity butanosolv lignin was achieved with carbohydrate impurities of 3% and a very high structural quality of 67 β-O-4 linking motifs. Mass-analysis showed that 69% of the carbohydrates were effectively removed in a single step caustic soda treatment step. In combination with a precipitation step a total carbohydrate removal of 98% was achieved, which was considerably higher than the 88% carbohydrate removal by the traditional work-up. The caustic soda treatment partially reverts part of the butoxylated carbohydrates back to their parent carbohydrates and also reduces the amount of butoxylated β-O-4 content in the lignin. Furthermore, the overall work-up scheme resulted in fractionation of butanosolv lignin in three fractions: 1) A low molecular weight (1900 DA) water soluble fraction that was readily recovered by acidification of the aqueous phase; 2) A main fraction (80+% of the total recovered lignin) of medium molecular weight lignin (3600 DA); 3) A residual high molecular weight lignin fraction (6900 Da).

These results show that the implementation of a caustic soda treatment step is highly beneficial for both the structural quality and the overall purity of the isolated butanosolv lignin in good yield. The removal of 69% of the carbohydrates in a single washing step could be further improved as a longer contact time or the simple inclusion of a second washing step (Schuur et al., 2010) might lead to further purified lignin. Furthermore, the use of solvents during the extraction or caustic soda treatment that are immiscible with water (e.g., 2-MeTHF (Stiefel et al., 2017; Smink et al., 2020)) could lead to further improvement of carbohydrate removal. Moving towards commercial implementation of this process, some technical points should be addressed. The use of very fine milled material can strain feasibility and thus the influence of the particle size of the biomass on the lignin extraction efficiency should be further tested. Additionally, efficient recovery of the extraction solvent and reuse of the applied acid in the extraction step and base in the purification step are key steps that should be assessed. Overall, this process could mean a significant step for the development of fully continuous biomass fractionation processes that yield lignin with a high C-O linking motif content that can be more readily processed to for example high value monomers by catalytic depolymerization.

## Data Availability

The original contributions presented in the study are included in the article/Supplementary Material, further inquiries can be directed to the corresponding author.
