# Supplementary material for: Highly Efficient Semi-Continuous Extraction and In-Line Purification of High β-O-4 Butanosolv Lignin
Source: Front Chem. 2021 May 10;9:655983. doi: 10.3389/fchem.2021.655983 (PMC8141753; doi:10.3389/fchem.2021.655983)
Supplement: Supplementary file 1 [file DataSheet1.pdf]

Highly efficient semi-continuous extraction and in-line purification of high β-O-4 butanosolv lignin

Douwe S. Zijlstra[1], Joren de Korte[1], Ernst P. C. de Vries[1], Lisanne Hameleers[2], Erwin Wilbers[1], Edita Jurak[2] and Peter J. Deuss[1*]

[1]Department of Chemical Engineering (ENTEG), University of Groningen, Groningen, The Netherlands

[2]Department of Bioproduct Engineering (ENTEG), University of Groningen, Groningen, The Netherlands

1. Methods

Flow-through setup

The flow-through system consist of a glass solvent reservoir (800 mL), pneumatic oscillatory pump (Williams; P250V225), pressure indicator (0-400 bar), GC oven (HP, 5890 series II), reactor (100 mL, cylindrical (length: 20 cm, diameter: 2,6 cm), SS 316L), Type K thermocouple with indicator, particulate filter (Swagelok, ¼ in. tube fitting, 2 micron pore size), adjustable back pressure regulator (Gommer B.V., BP-3 series, SS 316L) with pressure indicator (0-40 bar) and precision balance (Kern, PCB 2500-2). These units are connected by tubes and fittings (Swagelok, SS-316L, ½ and ¼ in.). Both openings of the reactor are closed with a glass filter (custom made, sintered, P2).

Continuous integrated mixer/separator (CINC)

The CINC, a 3535-B Arrowhead Drive (2002) has a max rpm of 6000, max throughput of 2 L/min, 200 g filling mass and 0-100 °C temperature range and is connected to the extraction liquor output with a 2 mm silicon tube.

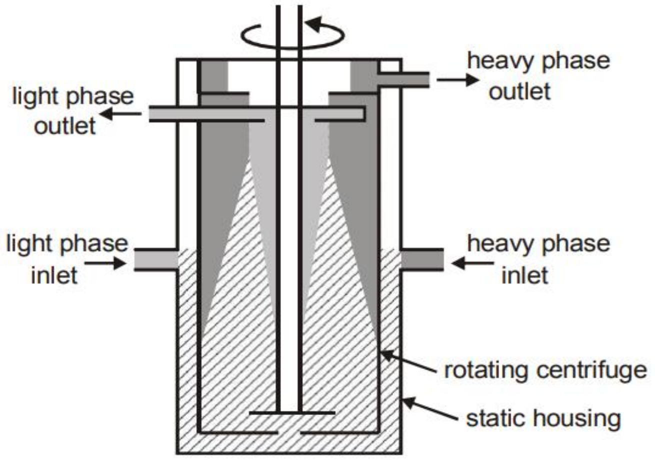

Figure S1: Schematic representation of the applied CINC setup (Schuur et al., 2008).

Synthesis of (2R/S,3R,4S,5R)-2-butoxytetrahydro-2H-pyran-3,4,5-triol (butyl xyloside): 10 g of D-xylose was heated at reflux conditions in a 90% n-BuOH/10% H_2O mixture containing 0.18 M H_2SO_4 for 2 hours. The reaction mixture was concentrated in vacuo to give the desired product as a viscous yellow oil which was characterized by NMR-spectroscopy without further purification.

2D Heteronuclear Single Quantum Coherence (HSQC) NMR spectroscopy: Structural analysis was performed by 2D HSQC NMR spectroscopy. 60 mg of butanosolv lignin was dissolved in 0.7 mL of d_6-acetone. A few drops of D_2O were added in order to ensure complete dissolution of the lignin. The NMR measurement was performed on a Bruker Ascend Neo 600 using the following parameters: (F2 = 11 to -1 ppm), (F1 = 160 to -10 ppm), nt = 4, ni = 512, d1 = 1.5, CNST [2] = 145 and the applied pulse sequence is hsqcetgpsi2. Analysis was

performed with Mestrenova. The obtained values for the detectable linking motifs were divided by a factor of 1.3 as previous work showed that the 2D HSQC NMR measurement overestimated these values (Zijlstra et al., 2019b).

^{31}P-NMR Spectroscopy: Hydroxyl content analysis was performed by ^{31}P-NMR spectroscopy using a literature procedure (Constant et al., 2016). 50 mg of lignin was dissolved in a 1.6:1 pyridine/$CDCl_3$ mixture (1.5 mL). 200 µL of an *endo-N*-hydroxy-5- norbornene-2,3-dicarboximide stock solution (9.2 mg/mL) was added as internal standard and 50 µL of a Chromium acetylacetonate stock solution (5.6 mg/mL) was added as relaxation agent. Afterwards, 100 µL 2-chloro-4,4,5,5-tetramethyl-1,3,2-dioxaphospholane was added as a derivatizing agent (Constant et al., 2016).

Gel Permeation Chromatography (GPC): The molecular weight of the isolated butanosolv lignin was determined by gel permeation chromatography (GPC) analysis. The analysis was carried out on a Hewlett Packard 1100 series THF-GPC. The lignin samples (10 mg) were dissolved in THF with toluene as a flow marker. Prior to analysis, the samples were filtered with a syringe filter (0.45 µm, PTFE). Analysis was performed with PSS WinGPC UniChrom.

Biomass analysis: Analysis of the carbohydrate and lignin content of the biomass sources and residual samples were performed following the NREL methodology (Sluiter et al. 2008). A 1,000 g dried raw biomass or residual biomass sample was added to a round bottom flask and 10 mL of a 72% aqueous H_2SO_4 was added. The resulting solution was incubated at 30 °C for 1 hour. After 1 hour, 280 mL of deionized water was added in order to obtain a 4% aqueous H_2SO_4 solution. The mixture was heated at reflux conditions for 1 hour and afterwards allowed to cool down to room temperature. Acid insoluble lignin was obtained by vacuum filtration and dried overnight in a vacuum oven at 60 °C. The amount of acid soluble lignin was determined by UV-VIS spectroscopy, using a background of a 4% sulfuric acid in deionized water. The samples were diluted in order to obtain an absorbance in the range of 0.7 – 1.0 at wavelength 240 nm.

The carbohydrate composition of the original material and the obtained residues (oven dried) was analyzed with HPAEC-PAD after acid hydrolysis (samples were diluted 20 - 50 times before injection). Therefore, 11 mg of each sample was mixed with 0,9 mL 72% (w/w) sulfuric acid and incubated for 1h at 30°C. After that 9.9 mL milliQ water was added, diluting the acid to 1 M. The incubation was continued for 3h at 100°C to fully hydrolyze the carbohydrates into monomers. All monomers were quantified by integrating the peak area of corresponding standards (arabinose, galactose, glucose, glucuronic acid, mannose, rhamnose, and xylose). Total sugar content was calculated as a sum of all neutral sugars and glucuronic acid .

High performance anion exchange chromatography (HPAEC) on a Dionex Ultimate 6000 system (Thermo Scientific, Sunnyvale, CA, USA) equipped with a CarboPac PA-1 column (2 mm x 250 mm ID) in combination with a CarboPac PA-1 guard column (2 mm x 50 mm ID) and PAD detection. System was controlled by the Chromeleon 7.2.9 software (Thermo Scientific, Sunnyvale, CA, USA). Elution of monosaccharides (0.25 mL min-1) was performed with a multi-step-gradient using the following eluents: A: 0.1M NaOH, B: 1M NaOAc in 0.1M NaOH, C: 0.2 M NaOH, and D: milliQ water. All analyzed monosaccharides elute in the first 20 min with 16% A, 84% D. Followed by 5 min with 45%A, 5%B, 50%D and 15 min with 60%A, 40% B. To regenerate the column it was flushed 12 min with 100% C by increasing flowrate in first 2 min to 0.35 mL min-1. Finally, the column is equilibrated for 12 min with 16%A, 84%D by decreasing the flowrate in the first 2 min to 0.25 mL min-1.

Source	Walnut powder		Beech wood		Reed		Douglas Fir		Pine wood	
	Starting material	Residual	Starting material	Residual	Starting material	Residual	Starting material	Residual	Starting material	Residual
**Lignin**	**40**	**8.4**	**25**	**1.8[a]**	**21.5**	**2.3[a]**	**24.9**	**23.6[a]**	**29.8**	**n.d.**
**Carbohydrate**	**28.2**	**7.3**	**22.4**	**1.4**	**38**	**2.2**	**38.3**	**6.2**	**39.9**	**1.0**
Xylose	*24.8*	*7.3*	*16.1*	*1.4*	*24.2*	*2.2*	*8.7*	*4.0*	*4.6*	*0.4*
Mannose	*0*	*0*	*0*	*0*	*0*	*0*	*15.7*	*2.2*	*5.9*	*0.6*
Galactose	*1.4*	*0*	*2.7*	*0*	*1.4*	*0*	*6.2*	*0*	*3.9*	*0*
Arabinose	*2.0*	*0*	*3.6*	*0*	*12.4*	*0*	*7.7*	*0*	*25.5*	*0*
**Glucose**	**26.2**	**83.6**	**47.1**	**86.3**	**39.5**	**71.4**	**43.0**	**73.0**	**31.6**	**42.9**
**Total**	**94.4**	**99.3**	**94.5**	**89.5**	**99**	**75.9**	**106.2**	**102.8**	**101.3**	**n.d.**

Table S1: Structural composition of the applied biomass (starting material) and the composition after organosolv extraction (residual). All values are given as a weight percentage (%w/w). [a)] values are calculated based on the values for the glucose in the starting material and the residual biomass (assuming no loss of glucose) and the values for the lignin extraction efficiency.

Method	β-O-4 content (per 100 C9 units)	β-β content (per 100 C9 units)	β-5 content (per 100 C9 units)	S/G/H ratio
Milled wood lignin	61	10	8	48/41/11
Enzymatic lignin	63	13	6	54/34/12

Table S2: Composition of walnut powder as determined by different analysis methodologies. Values are based on 2D HSQC NMR analysis.

**Particle size fraction (mm)**	**Weight percentage**
d > 0.425	0
0.300 < d < 0.425	49
0.212 < d < 0.300	41
d < 0.212	10

Table S3: Particle size distribution of walnut powder

Moisture content measurement: The moisture content of the biomass and the isolated lignin was determined with a PCE-MA 110 moisture meter. An ascension program to 120 °C was used during which the weight of the sample is tracked. At 120 °C the measurement is stopped if between 2 iterations the weight change is less than 0.2%. The obtained data was used to correct all yield calculations.

Sankey diagram: The Sankey diagram was made based on the following data and assumptions. The reported values between brackets were determined by the NREL analysis of the biomass. The other values are based on the determined mass of the isolated solid product (corrected for moisture content, butoxy-incorporation and carbohydrate impurities). It is assumed that no loss of product occurs in the separation steps. The reported value for lignin lost is the difference of the isolated lignin and the determined Klason Lignin content. The online tool SankeyMatic was used to generate the Sankey diagrams.

2. Comparison with other extraction procedures

Group	Conditions	Biomass source	Solvent efficiency (mg lignin extracted /gram solvent)
This work	9:1 nBuOH/H_2O<br>0.18 M H_2SO_4, 120 °C	Walnut	50
Bauer et al.	19:1 EtOH/H_2O<br>0.20 M HCl, 100 °C	Miscantus Giganteus	10
Lancefield et al.	19:1 nBuOH/H_2O<br>0.20 M HCl, 120 °C	Walnut	32
Luterbacher et al.	9:1 1,4-dioxane/formaldehyde<br>0.10 M HCl, 100 °C	Beech and transgenic Poplar	16
Huijgen et al.	1:1 Acetone/H_2O<br>0.015 M H_2SO_4, 190 °C	Beech, Poplar, Birch	19
Huijgen et al.	6:4 EtOH/H_2O<br>0.015 M H_2SO_4, 190 °C	Wheat Straw	13
Grande et al.	1:1 MeTHF/H_2O<br>0.05 M FDCA, 160 °C	Beech	18
Sun et al.	7:3 2-PrOH/H_2O<br>220 °C	Eucalyptus	20
Hensen et al.	MeOH<br>Varying acids, 160 °C	Oak sawdust	9
Ragauskas et al.	85:15 1,4-BDO/H_2O<br>0.015 M H_2SO_4, 170 °C	Eucalyptus	33
Bacher et al	12:44:44 SO_2/Alcohol/Water<br>155 °C	Sugarcane straw	47

Table S4: Solvent efficiency of a wide range of lignin extractions, all showing a lower solvent efficiency than our reported extraction procedure in this work. (Bauer et al., 2012; Wildschut et al., 2013; Shuai et al., 2016; Lancefield et al., 2017; Smit and Huijgen, 2017; Wang et al., 2017; Ouyang et al., 2018; Weidener et al., 2018; Dong et al., 2019)

3. Extraction and work-up flow schemes

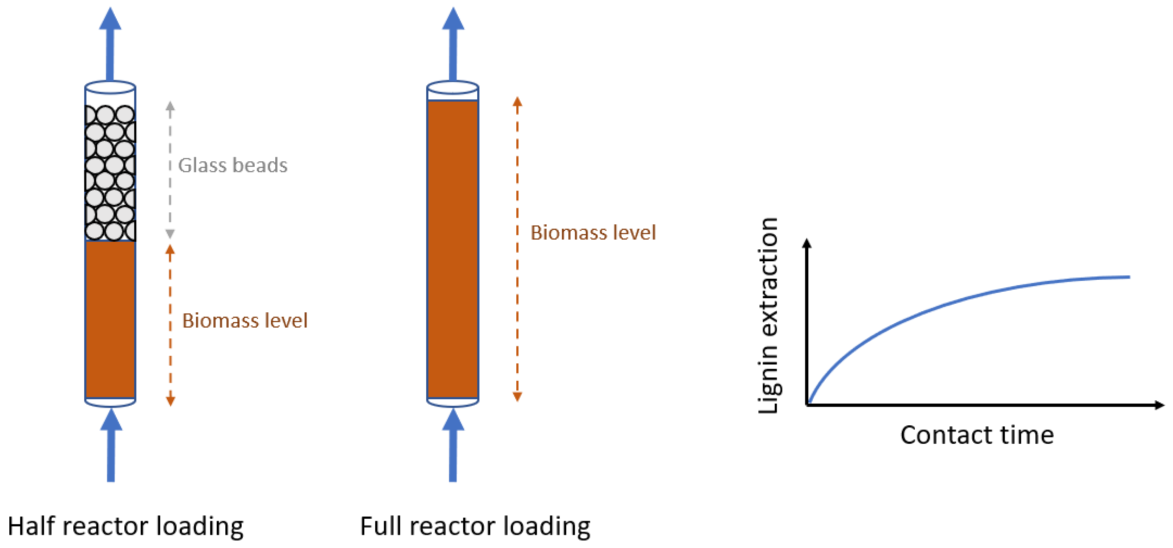

Figure S2: Schematic representation of the influence of the reactor loading on the biomass level. The small cylindrical tube used as reactor causes an increased contact time of the solvent with biomass, resulting in an increased lignin extraction.

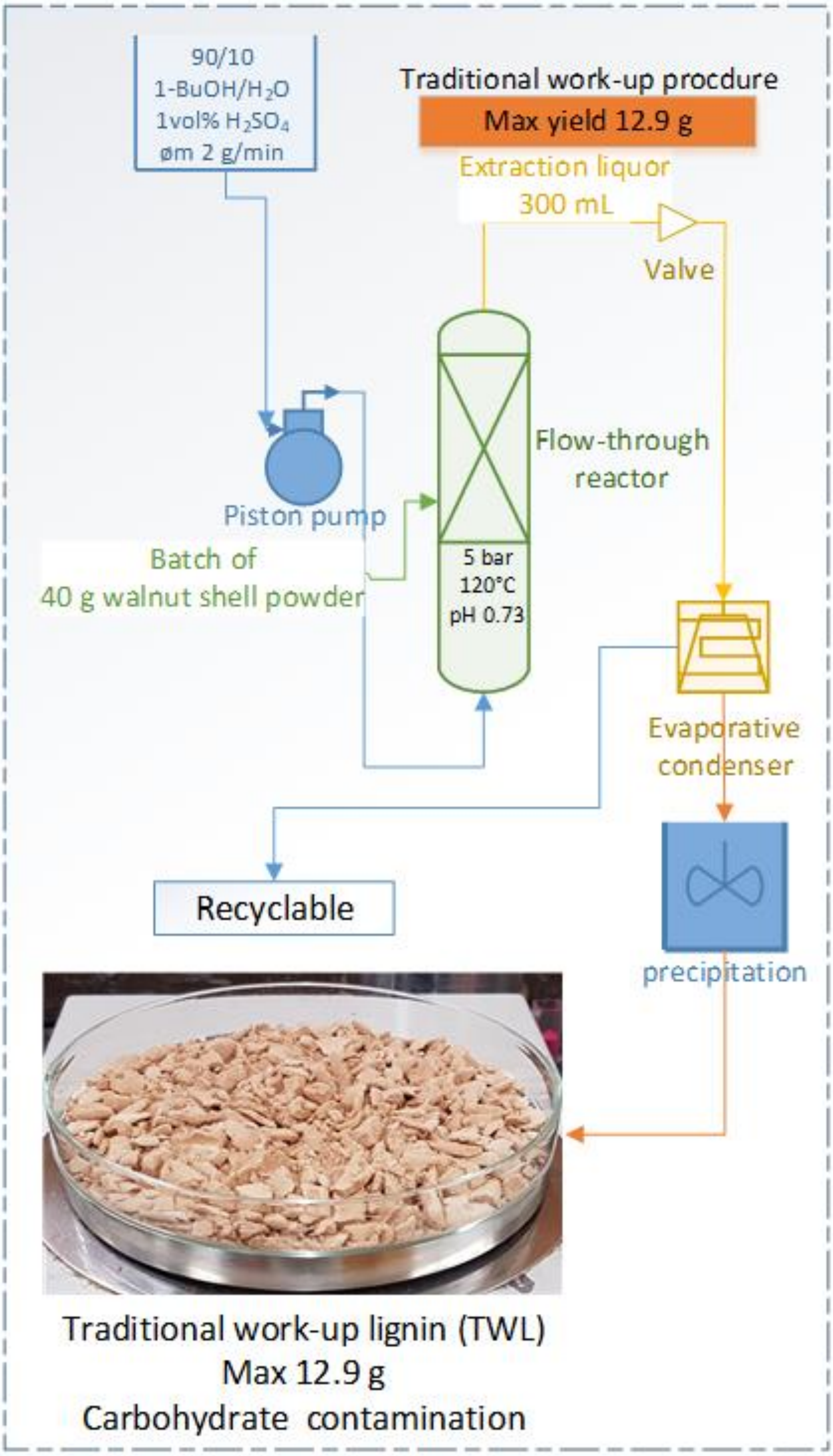

Figure S3: Schematic representation of the flow-schemes of flow-through extraction coupled with the traditional precipitation work-up procedure

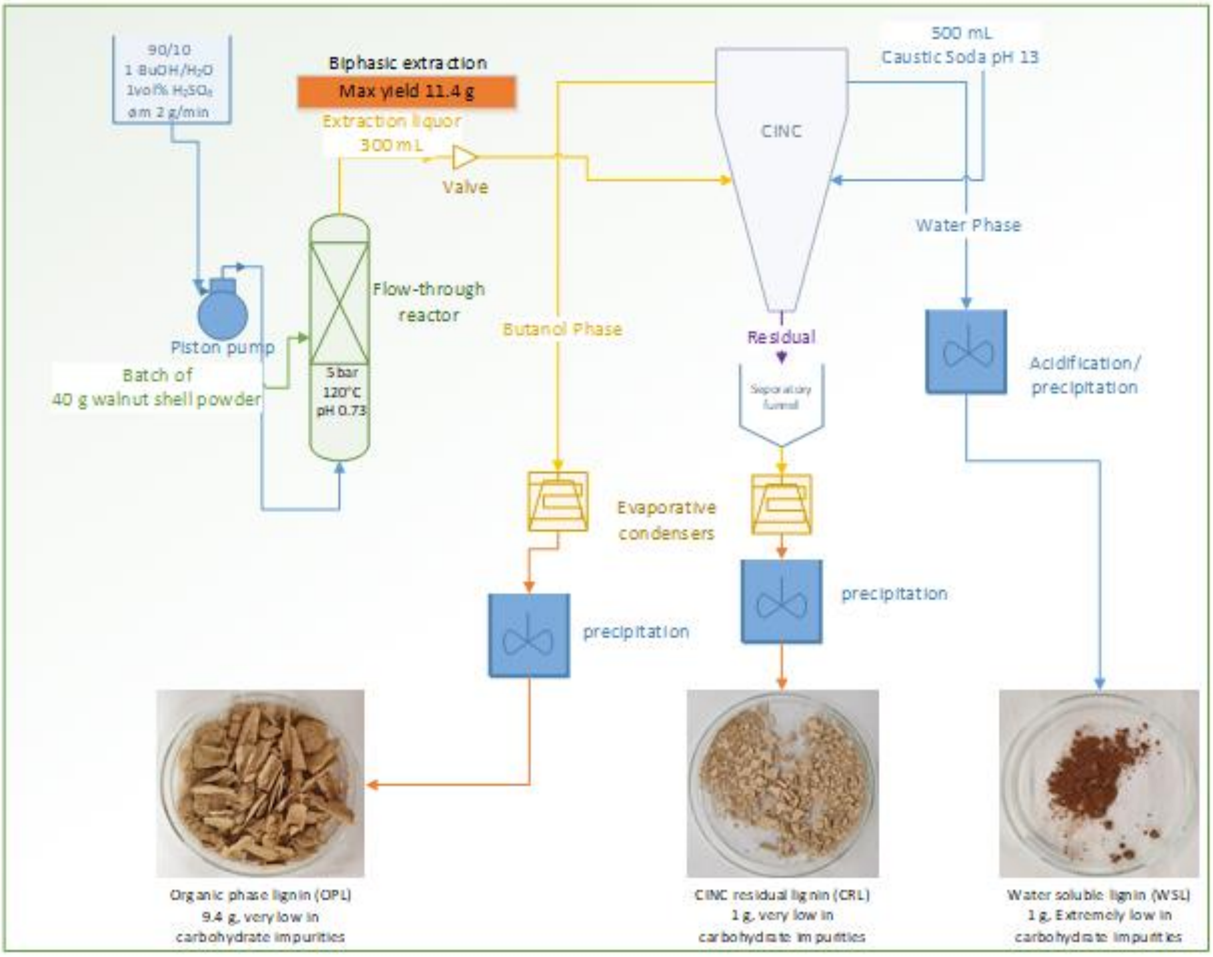

Figure S4: Schematic representation of the flow-schemes of flow-through extraction coupled with biphasic extraction and precipitation.

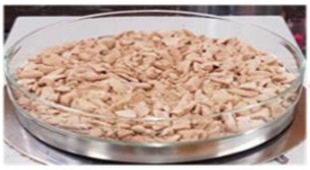

Traditional work-up lignin (TWL)
12.9 gram (corrected yield)
12% carbohydrate impurities

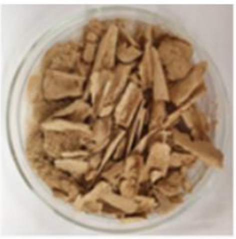

Organic phase lignin (OPL)
9.4 gram (corrected yield)
3% carbohydrate impurities

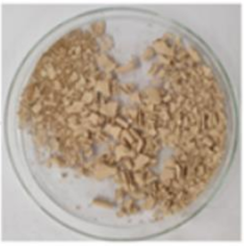

CINC residual lignin (CRL)
1.0 gram (corrected yield)
<1% carbohydrate impurties

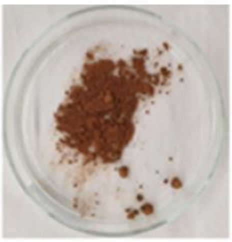

Water-soluble lignin (WSL)
1.0 gram (corrected yield)
<1% carbohydrate impurties

Figure S5: Isolated lignin obtained with the traditional work-up procedure (**top**) and the biphasic extraction + precipitation (**bottom**).

4. Traditional work-up issues

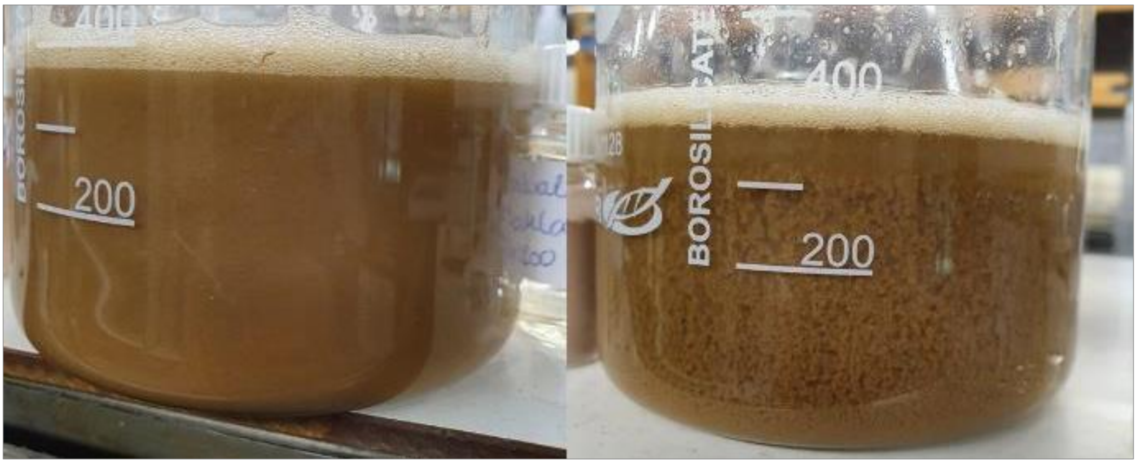

Figure S6: Precipitation of the concentrated butanosolv extraction liquor in water in the absence of sodium sulfate (left) and precipitation with sodium sulfate added to the water (right) which clearly enhances lignin flocculation.

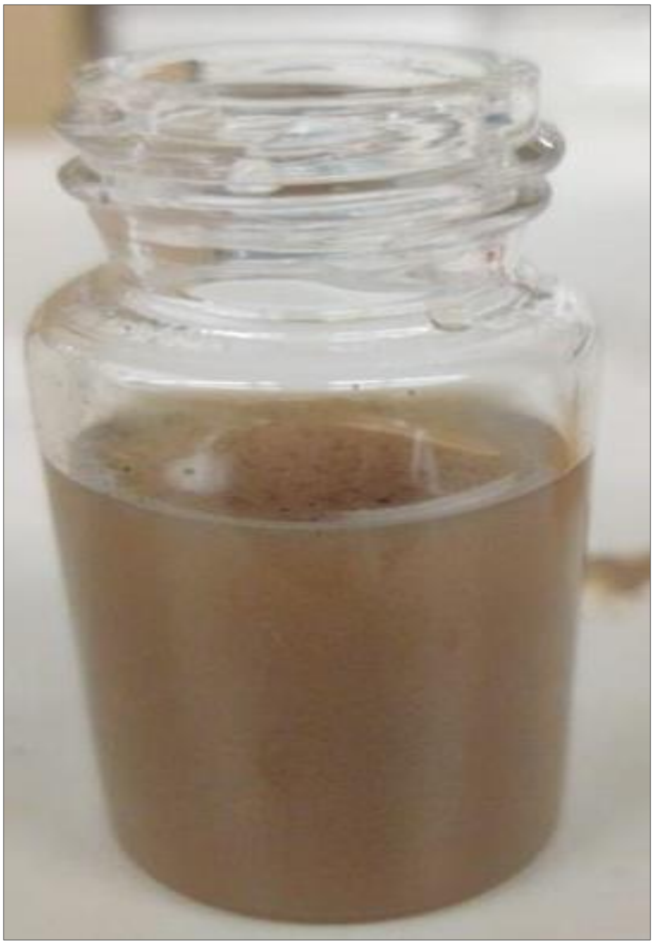

Figure S7: Solubility test of butyl-xyloside in water, clearly showing the mediocre solubility of modified xylose. During the lignin precipitation step part of the butyl-xyloside will also precipitate and be isolated as a solid product.

5. Butyl-xyloside determination

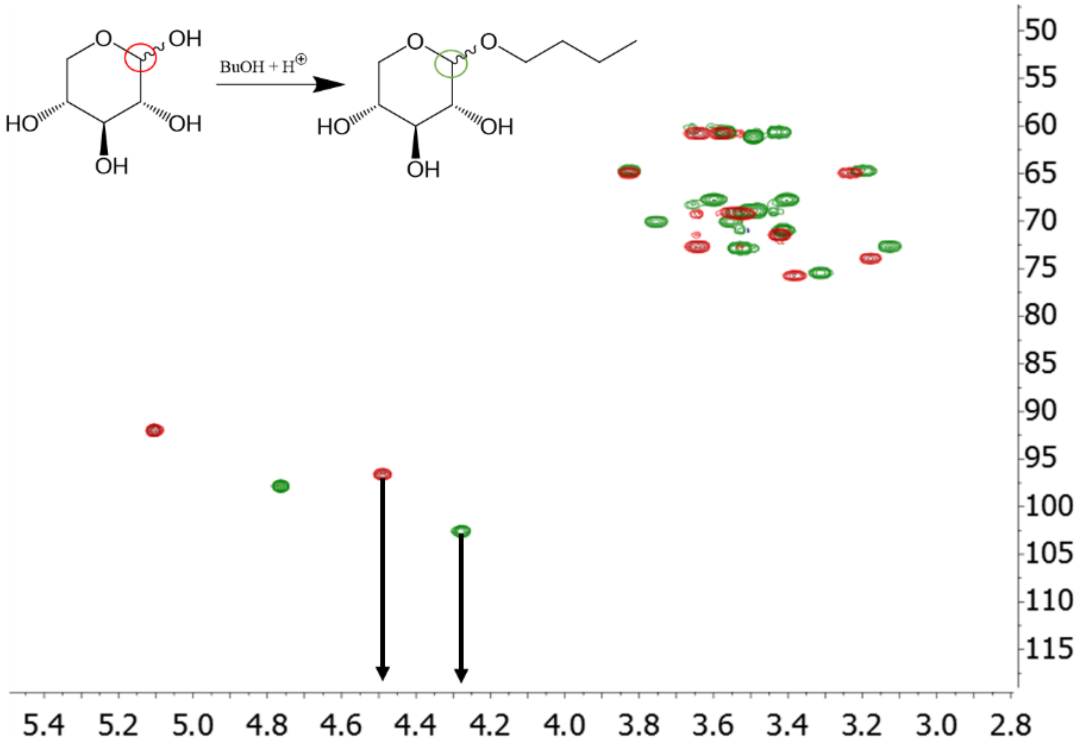

Figure S8: 2D-HSQC NMR (d_6-acetone) overlap of D-Xylose (red) and butyl-xyloside (green).

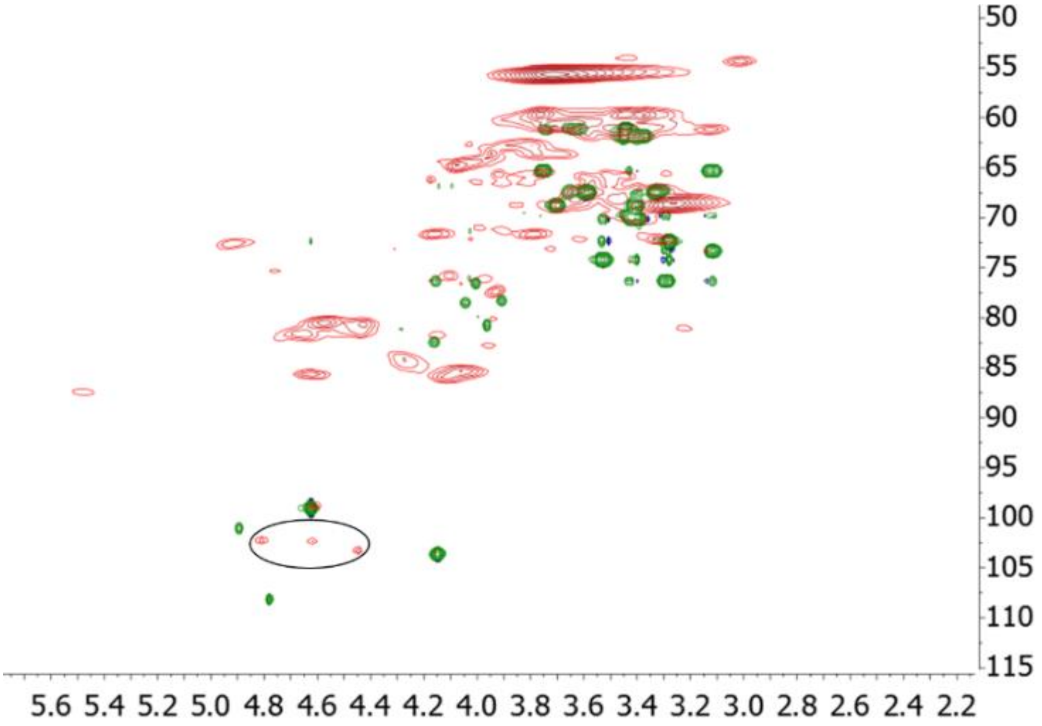

Figure S9: 2D-HSQC NMR overlap (d_6-acetone) of butyl-xyloside (green) and butanosolv lignin (red).

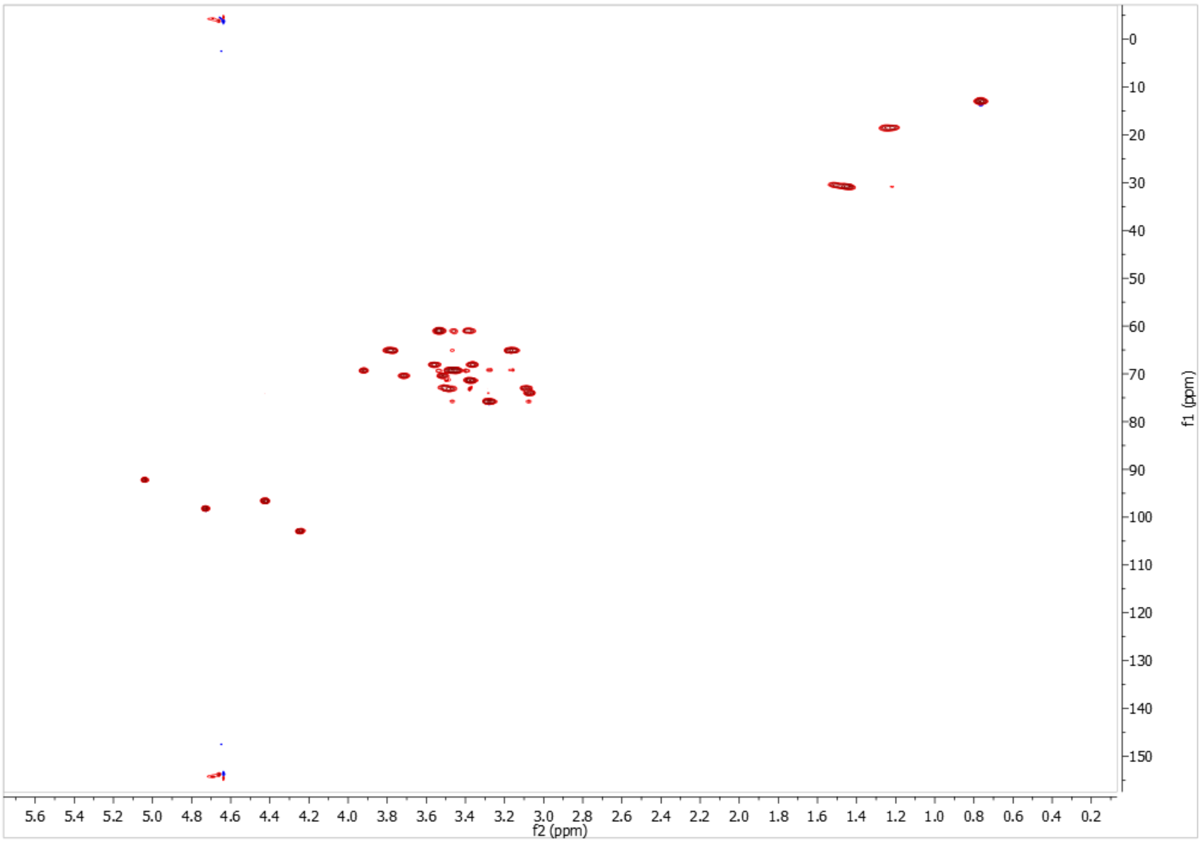

Figure S10: 2D HSQC NMR spectrum (d_6-acetone) of the product obtained after basic treatment of butyl-xyloside, showing partial reversion to D-xylose.

Sample	Aromatic -OH content	Aliphatic -OH content	Ratio Aromatic:Aliphatic
Butanosolv lignin	2.65	9.13	1:3.45
After caustic soda treatment	2.89	8.93	1:3.09

Table S5: Aromatic -OH and Aliphatic -OH content of butanosolv lignin and prior and after caustic soda treatment (5-substituted -OH was set at 1.00) as determined by ^{31}P-NMR analysis.

6. Molecular weight of lignin obtained by biphasic separation

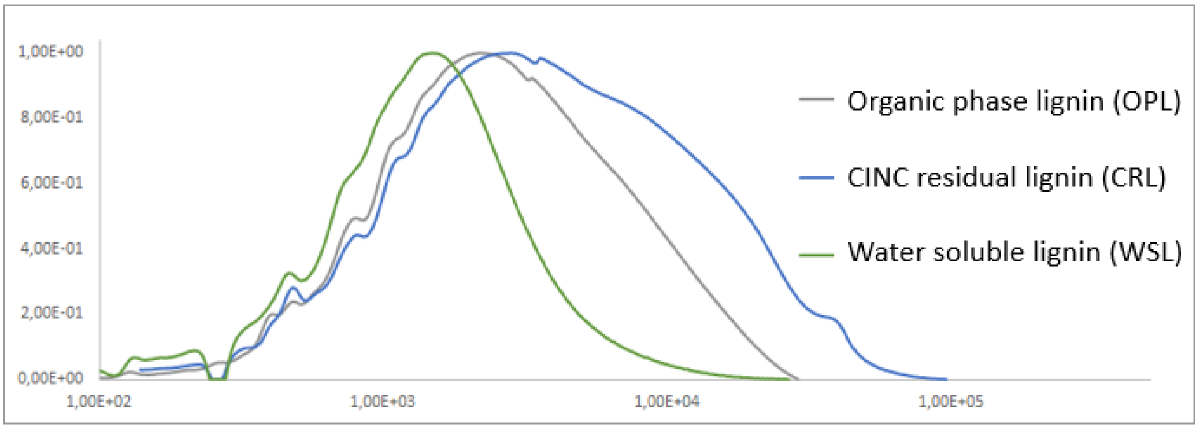

Figure S11: Molecular weight distribution of the organic phase lignin (OPL), Cinc residual lignin (CRL) and water soluble lignin (WSL) fraction after biphasic treatment determined by GPC.

Lignin type	Weight average molecular weight (Da)
Organic phase lignin	3900
CINC residual lignin	6900
Water Soluble lignin	1900

Table S6: Weight average molecular weight of the different lignin isolated with biphasic extraction.

7. Butanosolv pine wood extraction

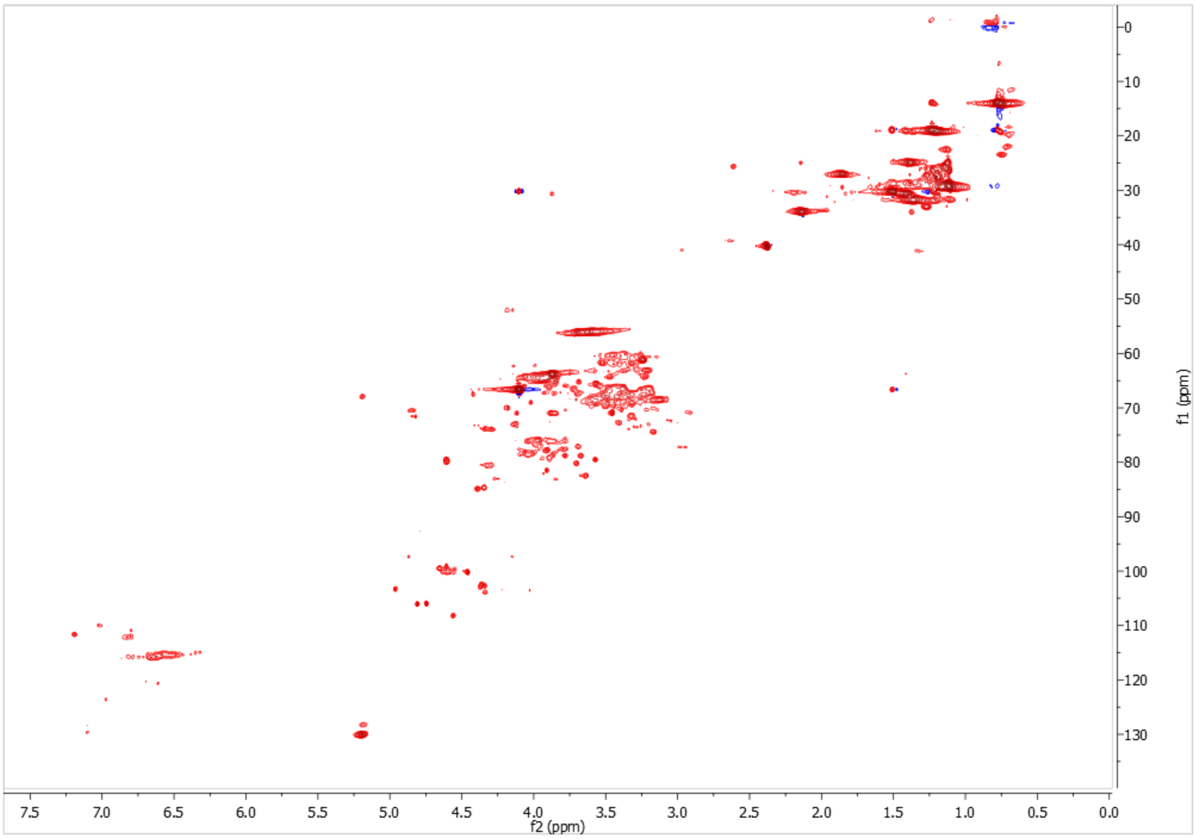

Figure S12: 2D HSQC NMR spectrum (d_6 acetone) of the product obtained after butanosolv extraction of pine wood.

8. Literature

Bauer, S., Sorek, H., Mitchell, V. D., Ibáñez, A. B., and Wemmer, D. E. (2012). Characterization of Miscanthus giganteus lignin isolated by ethanol organosolv process under reflux condition. *J. Agric. Food Chem.* 60, 8203–8212. doi:10.1021/jf302409d.

Constant, S., Wienk, H. L. J., Frissen, A. E., Peinder, P. de, Boelens, R., van Es, D. S., et al. (2016). New insights into the structure and composition of technical lignins: a comparative characterisation study. *Green Chem.* 18, 2651–2665. doi:10.1039/C5GC03043A.

Dong, C., Meng, X., Yeung, C. S., Tse, H. Y., Ragauskas, A. J., and Leu, S. Y. (2019). Diol pretreatment to fractionate a reactive lignin in lignocellulosic biomass biorefineries. *Green Chem.* 21, 2788–2800. doi:10.1039/c9gc00596j.

Lancefield, C. S., Panovic, I., Deuss, P. J., Barta, K., and Westwood, N. J. (2017). Pre-treatment of lignocellulosic feedstocks using biorenewable alcohols: Towards complete biomass valorisation. *Green Chem.* 19, 202–214. doi:10.1039/c6gc02739c.

Ouyang, X., Huang, X., Hendriks, B. M. S., Boot, M. D., and Hensen, E. J. M. (2018). depolymerization of woody biomass in a two-step. 2308–2319. doi:10.1039/c8gc00639c.

Schuur, B., Floure, J., Hallett, A. J., Winkelman, J. G. M., DeVries, J. G., and Heeres, H. J. (2008). Continuous chiral separation of amino acid derivatives by enantioselective liquid#liquid extraction in centrifugal contactor separators. *Org. Process Res. Dev.* 12, 950–955. doi:10.1021/op800074w.

Shuai, L., Amiri, M. T., Questell-Santiago, Y. M., Héroguel, F., Li, Y., Kim, H., et al. (2016). Formaldehyde stabilization facilitates lignin monomer production during biomass depolymerization. *Science (80-.).* 354, 329–333. doi:10.1126/science.aaf7810.

Sluiter, A., Hames, B., Ruiz, R., Scarlata, C., Sluiter, J., Templeton, D., et al. Determination of Structural Carbohydrates and Lignin in Biomass Determination of Structural Carbohydrates and Lignin in Biomass. *NREL/TP-510- 42618, US Natl. Renew. Energy Lab. Golden, Color. 2008*. doi:10.1007/s13398-014-0173-7.2.

Smit, A., and Huijgen, W. (2017). Effective fractionation of lignocellulose in herbaceous biomass and hardwood using a mild acetone organosolv process. *Green Chem.* 19, 5505–5514. doi:10.1039/c7gc02379k.

Wang, B., Shen, X. J., Wen, J. L., Xiao, L., and Sun, R. C. (2017). Evaluation of organosolv pretreatment on the structural characteristics of lignin polymers and follow-up enzymatic hydrolysis of the substrates from Eucalyptus wood. *Int. J. Biol. Macromol.* 97, 447–459. doi:10.1016/j.ijbiomac.2017.01.069.

Weidener, D., Klose, H., Leitner, W., Schurr, U., Usadel, B., de María, P. D., et al. (2018). One-step lignocellulose fractionation by using 2,5-furandicarboxylic acid as a biogenic and recyclable catalyst. *ChemSusChem* 11, 2051–2056. doi:10.1002/cssc.201800653.

Wildschut, J., Smit, A. T., Reith, J. H., and Huijgen, W. J. J. (2013). Ethanol-based organosolv fractionation of wheat straw for the production of lignin and enzymatically digestible cellulose. *Bioresour. Technol.* 135, 58–66. doi:10.1016/j.biortech.2012.10.050.
